# Pharmacological or genetic inhibition of hypoxia signaling attenuates oncogenic *RAS*-induced cancer phenotypes

**DOI:** 10.1242/dmm.048953

**Published:** 2021-11-19

**Authors:** Jun-yi Zhu, Xiaohu Huang, Yulong Fu, Yin Wang, Pan Zheng, Yang Liu, Zhe Han

**Affiliations:** 1 Center for Precision Disease Modeling, Department of Medicine, University of Maryland School of Medicine, Baltimore, MD 21201, USA; 2 Division of Immunotherapy, University of Maryland School of Medicine, Baltimore, MD 21201, USA; 3 Division of Endocrinology, Diabetes and Nutrition, Department of Medicine, University of Maryland School of Medicine, Baltimore, MD 21201, USA

**Keywords:** *Drosophila*, Mouse xenografts, Leukemia, Oncogenic *RAS*, Echinomycin, Hypoxia pathway, HIF1A

## Abstract

Oncogenic Ras mutations are highly prevalent in hematopoietic malignancies. However, it is difficult to directly target oncogenic RAS proteins for therapeutic intervention. We have developed a *Drosophila* acute myeloid leukemia model induced by human *KRAS^G12V^*, which exhibits a dramatic increase in myeloid-like leukemia cells. We performed both genetic and drug screens using this model. The genetic screen identified 24 candidate genes able to attenuate the oncogenic *RAS*-induced phenotype, including two key hypoxia pathway genes *HIF1A* and *ARNT* (*HIF1B*). The drug screen revealed that echinomycin, an inhibitor of HIF1A, can effectively attenuate the leukemia phenotype caused by *KRAS^G12V^*. Furthermore, we showed that echinomycin treatment can effectively suppress oncogenic *RAS*-driven leukemia cell proliferation, using both human leukemia cell lines and a mouse xenograft model. These data suggest that inhibiting the hypoxia pathway could be an effective treatment approach and that echinomycin is a promising targeted drug to attenuate oncogenic *RAS*-induced cancer phenotypes.

This article has an associated First Person interview with the first author of the paper.

## Abstract (precis)

**Summary:** Hypoxia pathway inhibition, either genetically or pharmacologically, rescues *RAS*-induced oncogenesis in a *Drosophila* acute myeloid leukemia model, mouse xenograft model and human leukemia cells.

## INTRODUCTION

Mutations of the *RAS* family genes, consisting of *HRAS*, *KRAS* and *NRAS*, are involved in over 30% of all human cancers (Downward, 2015; Pylayeva-Gupta et al., 2011). *KRAS* is an especially prevalent contributor to human cancers affecting the pancreas, colon and lung (Downward, 2015; Pylayeva-Gupta et al., 2011). *RAS* mutations have also been associated with different types of leukemias, including acute myeloid leukemia (AML) (Beaupre and Kurzrock, 1999a,b; Braun and Shannon, 2008; Liu et al., 2019).

*RAS* genes encode highly conserved GTP-binding proteins involved in eukaryotic cell proliferation and differentiation (McCormick, 1994), which cycle between active GTP-bound and inactive GDP-bound states. Activated RAS, in turn, activates downstream effector pathways. Many oncogenic *RAS* mutations lock RAS in a constitutively activated state, which triggers the activation of multiple interacting signaling pathways (Bos, 1989; Downward, 2003). Activated RAS promotes cellular transformation through molecular signaling that stimulates cell proliferation, inhibits differentiation, blocks cell death and promotes angiogenesis (Hanahan and Weinberg, 2000). To date, efforts selectively and directly targeting oncogenic RAS have been extremely challenging. Although a few direct inhibitors for oncogenic RAS have been developed recently, they have shown variable responsiveness. Thus, invigorating the call for combinatorial strategies to optimize treatment of *RAS*-mediated cancers has been proposed (Hallin et al., 2020).

The *KRAS^G12V^* allele is one of the most prevalent oncogenes found in human cancers, and has been the focus of many previous studies to identify candidate genes or pathways representing potential therapeutic targets (Barbie et al., 2009; Corcoran et al., 2013; Kim et al., 2013; Luo et al., 2009; Sarthy et al., 2007; Scholl et al., 2009; Singh et al., 2012; Steckel et al., 2012; Vicent et al., 2010). However, seemingly promising candidates identified through *in vitro* genetic screens have failed to translate into clinically promising therapeutic targets. They often stumble at the *in vivo* validation phase, with the *in vitro* findings unable to be replicated in animal models and human patients (Downward, 2015). The translation hurdle urges the need for oncogenic RAS genetic screens and drug screens using *in vivo* lower-animal models that accommodate large-scale screens, such as *Drosophila*, followed by validation studies in human cells and mammalian animal models, such as mice.

Studies in *Drosophila* have proven extremely valuable for elucidating key features of *RAS* cellular physiology, and pathways affected by RAS protein activation are highly conserved from flies to humans (Asha et al., 2003; Brumby and Richardson, 2003, 2005; Gonzalez, 2013; Lusk et al., 2017; Ma et al., 2017; Pagliarini and Xu, 2003; Vidal and Cagan, 2006; Wu et al., 2010). *Drosophila* possesses a single *Ras85D* gene, in contrast to the human *KRAS*, *NRAS* and *HRAS* family members. Although genetic screens have been conducted previously for fly cancer models with oncogenic mutations in the fly *Ras85D* gene (Asha et al., 2003; Chabu and Xu, 2014; Gladstone and Su, 2011; Ma et al., 2017; Pagliarini and Xu, 2003), no previous studies have been described that use a fly cancer model induced by a human oncogenic *RAS* mutation.

In this study, we established a new *Drosophila* cancer model induced by expressing human *KRAS^G12V^* in fly hemocytes. This model exhibits a dramatic increase in hemocyte numbers caused by overproliferation, which provides an ideal phenotype for genetic and drug screens. Using this model in a genetic screen, we identified 24 promising hits that, when silenced in the hemocytes, attenuated the leukemia phenotype of the *KRAS^G12V^* model, including two key components of the hypoxia pathway, *HIF1A* and *ARNT* (*HIF1B*), which are involved in the development of both solid tumors and hematological malignancies (Deynoux et al., 2016; Jun et al., 2017; Semenza, 2003; Szymczak et al., 2018). A drug screen using the same model revealed that echinomycin, an inhibitor of HIF1A, could antagonize the *KRAS^G12V^*-induced leukemia phenotype. We validated this finding in human leukemia cell lines as well as a mouse xenograft model, demonstrating the promising translational value of this finding and expanding its application to a broad range of oncogenic *RAS* mutations.

## RESULTS

### Generation of a new *KRAS^G12V^*-induced *Drosophila* leukemia model

**Fig. 1.**
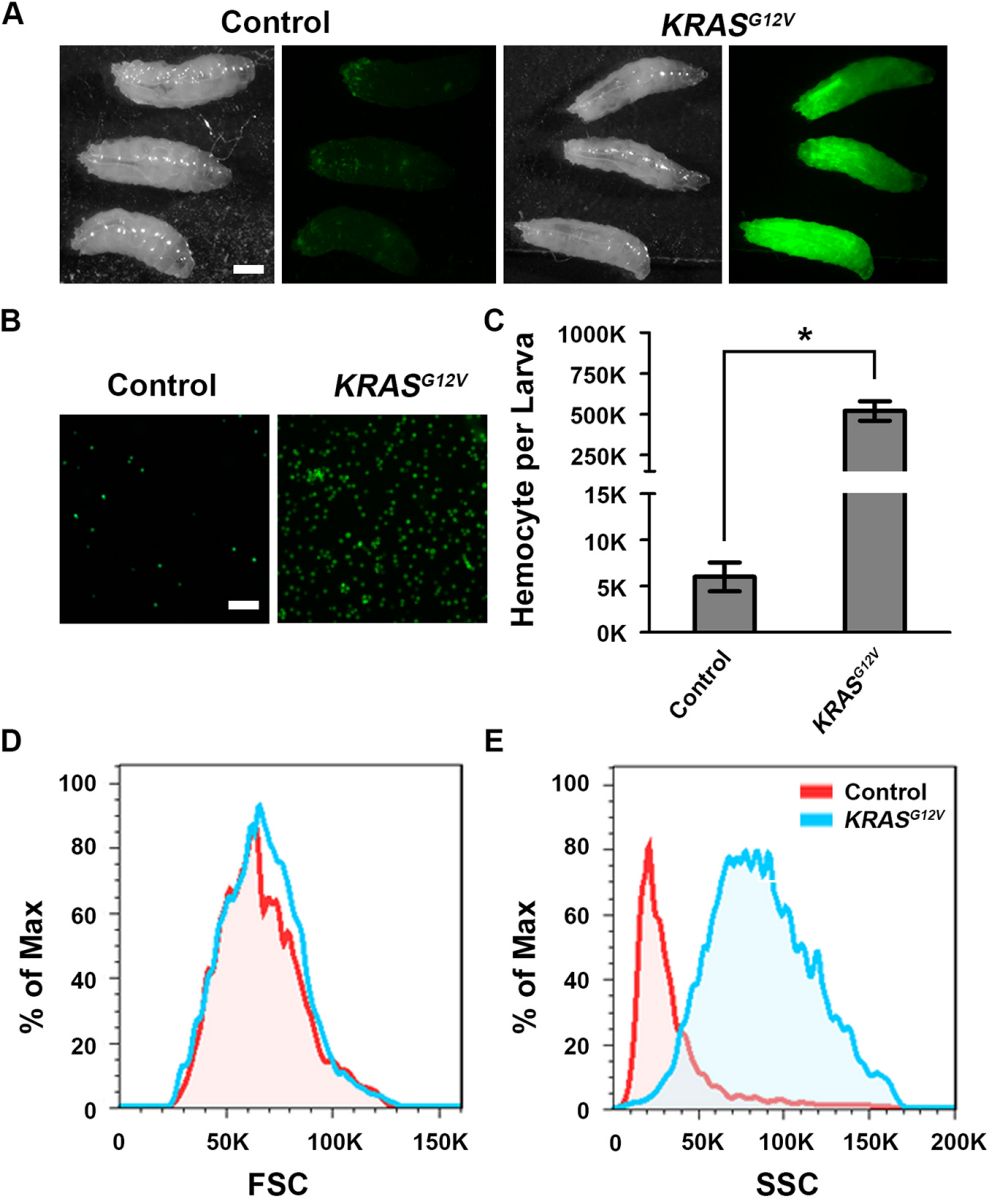
**Human *KRAS^G12V^* mutant transgene drives abnormal hemocyte proliferation in a *Drosophila* leukemia model.** (A) Transgenic third-instar larvae carrying hemocyte-specific *Hml-Gal4* driver directing expression of *UAS-GFP* (Control), and *UAS-GFP* plus *UAS-HsKRAS^G12V^* (*KRAS^G12V^*). Panels show stereo micrographs of larvae (left) and GFP fluorescence micrographs (right). Scale bar: 0.3 mm. (B) Hemocytes in hemolymph samples of equal volume extracted from control and *KRAS^G12V^* third-instar larvae. Scale bar: 50 μm. (C) Quantification of total hemocytes per control and *KRAS^G12V^* third-instar larvae (*n*=6; results are presented as mean±s.d.; *P<0.05; unpaired Student's *t*-test). (D) Frequency plots comparing control and *KRAS^G12V^* third-instar larval hemocyte cell size. Forward scatter (FSC) is a measurement of the amount of the laser beam that passes around the cell, which gives a relative size of a cell. (E) Frequency plots comparing control and *KRAS^G12V^* third-instar larval hemocyte cell structure. Side scatter (SSC) is a measurement of the amount of the laser beam that bounces off particulates inside the cell, which is an indicator of granularity in a cell.

We generated a new *UAS*-*KRAS^G12V^* transgenic line to express the oncogenic human *KRAS^G12V^* allele in different types of tissues. When we used *Hml*-*Gal4* to express *KRAS^G12V^* (alone with *UAS*-*GFP* as a marker) in the *Drosophila* hemocytes, we noticed a dramatic increase in GFP^+^ hemocytes (Fig. 1A). At 25°C, third-instar larvae with *Hml*-*Gal4*-driven *UAS*-*KRAS^G12V^* (genotype *Hml*-*Gal4, UAS*-*GFP; UAS*-*KRAS^G12V^*) contained nearly 100-fold more circulating hemocytes than the control (genotype *Hml*-*Gal4, UAS*-*GFP*) flies (Fig. 1B,C). Using a flow-cytometry equipment with high-throughput imaging function, we analyzed the size and granularity of hemocytes in the control and *KRAS^G12V^* flies (Fig. 1D,E). We found that the size of *KRAS^G12V^* hemocytes did not change significantly (Fig. 1D); however, the hemocytes exhibited significantly increased intracellular granularity (Fig. 1E).

### *KRAS^G12V^* increases the percentage of Wg-expressing and Lz-expressing hemocytes, and decreases P1-labeled matured hemocytes

**Fig. 2.**
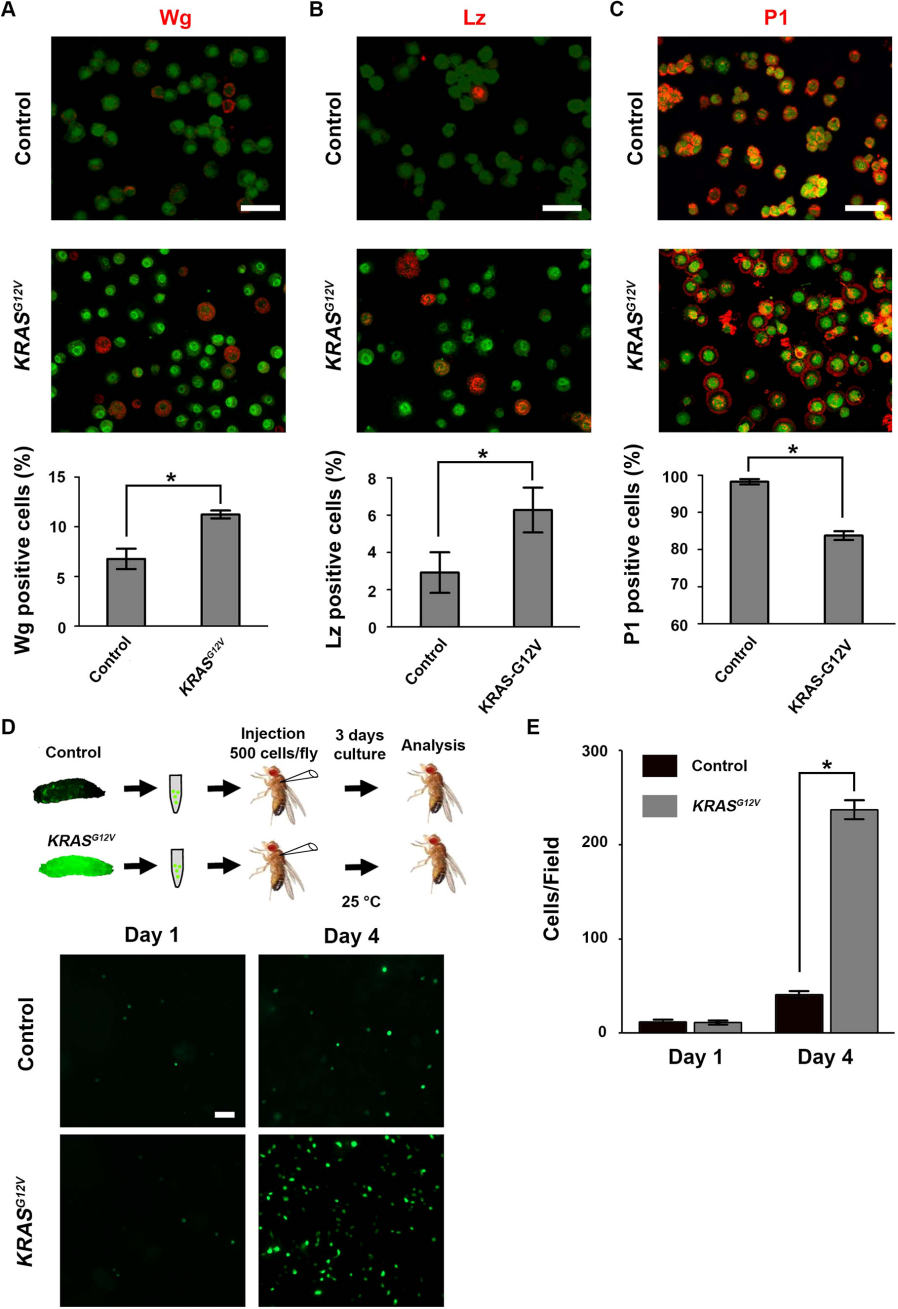
**Human *KRAS^G12V^* transgene expression alters the distribution of hemocyte subtypes and drives abnormal cell population expansion after transplantation.** (A) Pro-hemocytes were selectively immunolabeled with anti-Wingless (Wg) antibody. The percentage of pro-hemocytes in total hemocyte is increased ∼1.5- to 2-fold in *KRAS^G12V^* larvae (quantification shown below; *n*=6; results are presented as mean±s.d.; *P<0.05; unpaired Student's *t*-test). Scale bar: 30 μm. (B) Crystal cells were selectively immunolabeled with anti-Lozenge (Lz) antibody. The percentage of crystal cells in total hemocytes is increased ∼2-fold in *KRAS^G12V^* larvae (quantification shown below; *n*=6; results are presented as mean±s.d.; *P<0.05; unpaired Student's *t*-test). Scale bar: 30 μm. (C) Plasmatocytes were selectively immunolabeled with anti-P1 antibody. The percentage of plasmatocytes in total hemocytes is reduced ∼10% in hemocytes *KRAS^G12V^* larvae (quantification shown below; *n*=6; results are presented as mean±s.d.; *P<0.05; unpaired Student's *t*-test). Scale bar: 30 μm. (D) Schematic showing larval hemocyte transplantation into adult flies. Hemocytes were collected from control (*Hml-Gal4, UAS-GFP*) or *KRAS^G12V^* (*Hml-Gal4, UAS-GFP; UAS-KRAS^G12V^*) third-instar larvae, then injected (500 hemocytes) into adult flies. Injected flies were maintained for 4 days, at the end of which time the hemolymph was collected and analyzed microscopically for hemocyte density. Lower panels show fluorescence micrographs of control compared to *KRAS^G12V^* hemocytes (expressing GFP) 1 day after transplantation (day 1) and day 4 post-transplantation into adult flies. Scale bar: 50 μm. (E) Quantification of transplanted hemocyte numbers at day 1 and day 4 post-transplantation (results are presented as mean±s.d.; *P<0.05; unpaired Student's *t*-test).

The *Drosophila* hematopoietic system consists of different cell types that undergo different stages of differentiation (Evans et al., 2014; Fu et al., 2020). We analyzed the relative proportions of hemocyte subtypes that express signature differentiation markers, such as Wingless (Wg), Lozenge (Lz) and Nimrod C1 (Fig. 2), to determine the effects of *KRAS^G12V^* on hemocyte differentiation. We found that *KRAS^G12V^* induced increased numbers of Wg-expressing hemocytes (Fig. 2A), which has been associated with stem-like hemocyte precursors because Wg expression withdraws as hemocytes differentiate (Sinenko et al., 2009). We found that Wg-expressing hemocytes also exhibited weaker *Hml*-*Gal4*-driven GFP expression (Fig. 2A). We also found that crystal cells with Lz expression increased their proportion in *KRAS^G12V^* flies (Fig. 2B). In contrast, plasmatocyte expression of Nimrod C1, a terminally differentiated plasmatocyte marker that is recognized by the anti-P1 antibody (Kurucz et al., 2007), was significantly reduced in proportion in the *KRAS^G12V^* flies (Fig. 2C).

### *KRAS^G12V^*-induced hemocyte overproliferation is cell autonomous

We next tested whether *KRAS^G12V^*-induced overproliferation of hemocytes was cell autonomous using a leukemia cell transplant assay. We collected GFP^+^ hemocytes from the *KRAS^G12V^* leukemia fly model donor, in parallel with the control donor (*Hml*-*Gal4, UAS*-*GFP*). After careful titrating, we injected ∼500 GFP^+^ donor hemocytes into wild-type (*w*^1118^) flies and followed up by counting GFP^+^ hemocytes in transplant recipients at two successive time points. The number of GFP^+^ hemocytes did not change significantly in the first few hours after transplantation (Fig. 2D, Day 1). However, after 3 days (Day 4 in Fig. 2D), GFP^+^ hemocytes from the *KRAS^G12V^* donors were ∼20-fold more abundant than GFP^+^ hemocytes from the control donors (Fig. 2D,E), suggesting that *KRAS^G12V^*-induced hemocyte overproliferation is cell autonomous.

### *KRAS^G12V^* compromises the immune function of hemocytes in leukemia flies

**Fig. 3.**
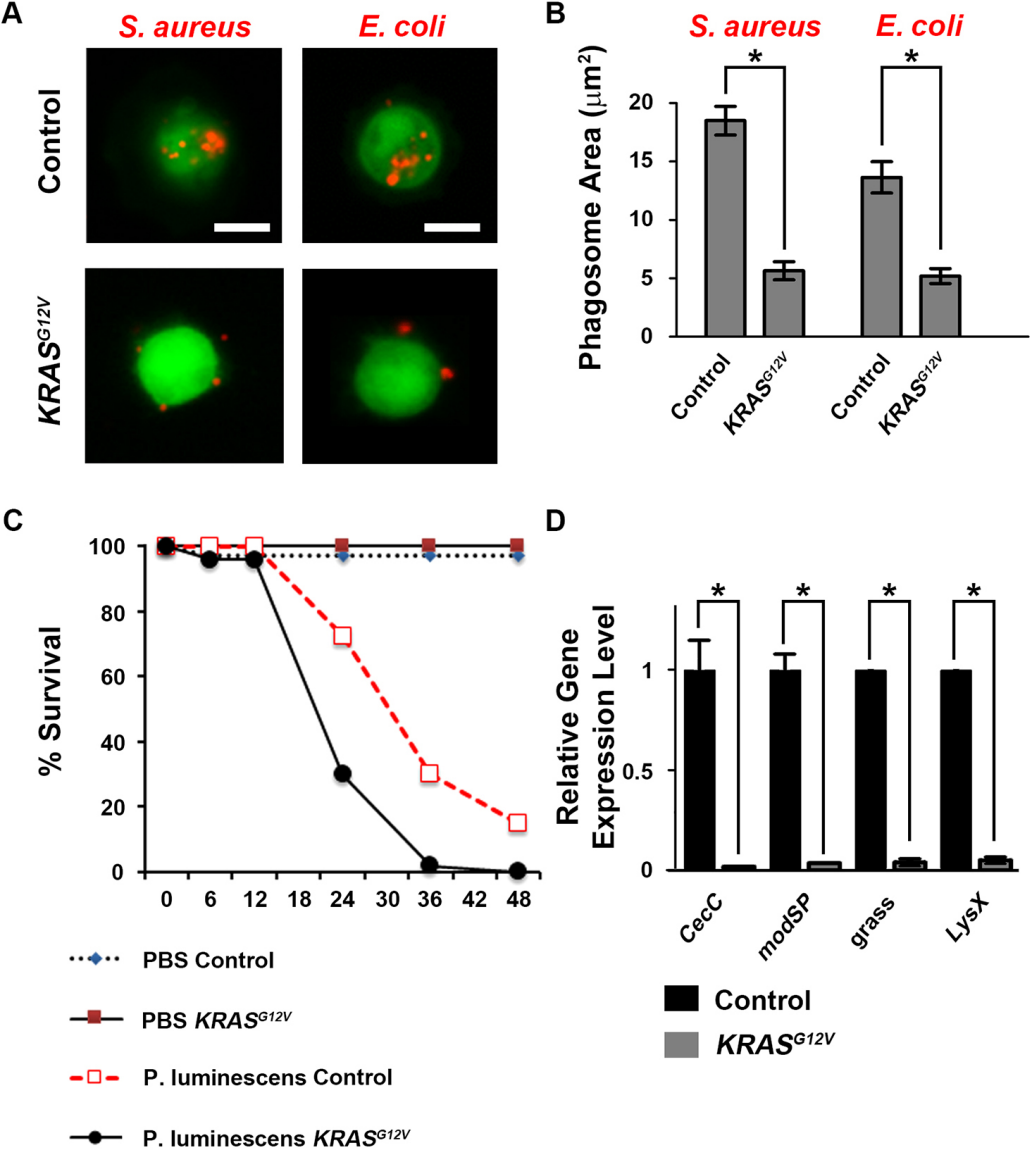
**Human *KRAS^G12V^* transgene expression is associated with *Drosophila* hemocyte immune function deficits.** (A) Hemocytes (green) from control (*Hml-Gal4, UAS-GFP*) and *KRAS^G12V^* (*Hml-Gal4, UAS-GFP; UAS-KRAS^G12V^*) third-instar larvae co-incubated with fluorescent pHrodo Red-tagged *S. aureus* or *E. coli* (red). Scale bars: 10 μm. (B) Quantification of phagosome area in control and *KRAS^G12V^* hemocytes co-incubated with fluorescent pHrodo Red-tagged *S. aureus* or *E. coli* (*n*=100; results presented as mean±s.d.; *P<0.05; unpaired Student's *t*-test). (C) Survival curves of control and *KRAS^G12V^* adult flies infected with pathogenic *P. luminescens* bacteria or sterile PBS (vehicle) at 18°C. Forty flies were analyzed for each group. (D) Quantitative RT-PCR showing the relative expression level of genes involved in antibacterial defense in hemocytes from control compared to *KRAS^G12V^* flies (*n*=3 replicates in each group; results presented as mean±s.d.; *P<0.05; unpaired Student's *t*-test). CecC, Cecropin C; modSP, modular serine protease; grass, Gram^+^-specific serine protease; LysX, Lysozyme X.

Human leukemia patients are at increased risk of suffering mortality as a result of infection due, at least in part, to immune cell dysfunction associated with transformation (Lamble and Lind, 2018). Hemocytes are critical components of the *Drosophila* innate immune system, and we observed that *KRAS^G12V^* expression led to reduced numbers of the P1-expressing plasmatocytes that are largely responsible for phagocytosis of bacteria (Govind, 2008). We therefore analyzed the immune functional capabilities of *KRAS^G12V^*-expressing hemocytes. *Drosophila* immunity can distinguish between Gram^+^ and Gram^−^ bacteria through different pathways. First, we tested the ability of larval hemocytes to phagocytose these two different types of bacteria: Gram^+^ bacteria *Staphylococcus aureus* and Gram^−^ bacteria *Escherichia coli*, each tagged with the fluorescent label pHrodo Red. Whereas normal hemocytes readily phagocytosed both bacterial species, *KRAS^G12V^* hemocytes showed significantly reduced phagocytic activity for both the Gram^+^ bacteria *S. aureus* and the Gram^−^ bacteria *E. coli* (Fig. 3A,B), even though the percentage of *KRAS^G12V^* hemocytes displaying phagocytic activity was not reduced (Fig. S1).

We also tested overall immune function by infecting adult flies with pathogenic *Photorhabdus luminescens* bacteria, which are known to suppress the insect immune response and even cause lethality to healthy flies. Because flies expressing *KRAS^G12V^* showed lethality at the pupal stage at 25°C, we maintained the *Hml*-*Gal4*, *UAS-GFP; UAS-KRAS^G12V^* leukemia model at 18°C to obtain adult flies for injection. We found that *KRAS^G12V^* still induced significant overproliferation of hemocytes at 18°C, but not as dramatically as at 25°C (Fig. S2). The mortality rates of the control and *KRAS^G12V^* flies were not affected by injection of phosphate buffered saline (1× PBS), demonstrating that injection alone did not harm the flies. When injected with same volume of *P. luminescens* in 1× PBS, the *KRAS^G12V^* flies exhibited significantly decreased resistance to the infection. At 24 h after infection, only ∼30% of *KRAS^G12V^* flies remained alive compared to over 70% of control flies (Fig. 3C). Finally, using RT-PCR, we examined the hemocyte expression levels of four genes (*CecC*, *modSP*, *grass* and *LysX*) that have been previously associated with defense against both Gram^−^ and Gram^+^ bacteria in *Drosophila* (Arefin et al., 2017). We found that *KRAS^G12V^* expression was associated with dramatically reduced expression of these antibacterial response genes in hemocytes (Fig. 3D). Overall, our data suggested that *KRAS^G12V^*-induced overproliferation of hemocytes leads to significantly compromised immunity and increased susceptibility to infection.

### A genetic screen in *Drosophila* to identify modifiers of the *KRAS^G12V^*-induced hemocyte overproliferation phenotype

**Fig. 4.**
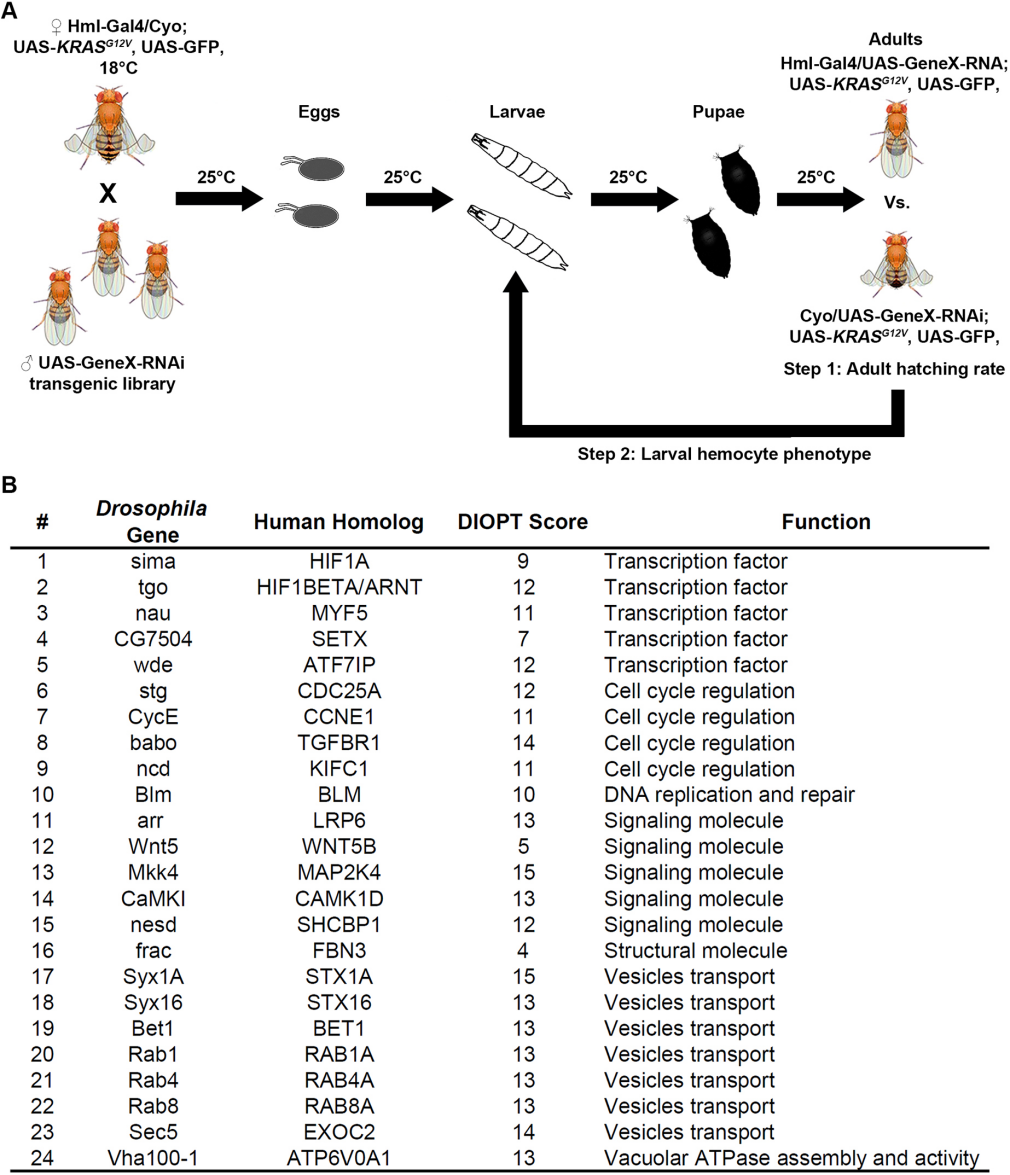
***Drosophila KRAS^G12V^* genetic screen and the hits that rescue the hemocyte overproliferation and developmental lethality phenotypes.** (A) Schematic illustration of the approach used for the genetic screen (see Materials and Methods section for a detailed description of rationale, strategy and procedure). (B) The 24 *Drosophila* genes that, when silenced in hemocytes, completely rescued *KRAS^G12V^*-induced hemocyte overproliferation and developmental lethality phenotypes. Also listed are the human homologs of these genes, their DIOPT score (indicating the degree of conservedness) and the predicted function of the proteins encoded by these genes.

The discovery that our *KRAS^G12V^* fly leukemia model can survive to adult stage at 18°C, but not at 25°C (owing to Gal4 being more stable at 25°C and hence driving higher levels of *KRAS^G12V^* expression), allowed us to design a high-throughput genetic screen to identify the genes that, when silenced in the hemocytes, attenuate the adult lethality of the *KRAS^G12V^* fly leukemia model at 25°C (Fig. 4A). We designed this screen to be performed in two steps. Step 1 is a high-throughput one-step cross to compare the number of adult fly progenies with straight-wing (*Hml*-*Gal4; UAS*-*KRAS^G12V^. UAS*-*GeneX-*RNAi) versus curly-wing (control, with only the *UAS*-*KRAS^G12V^. UAS*-*GeneX-*RNAi but not *Hml*-*Gal4*) phenotypes. The ratio will be 1:1 if silencing of *GeneX* prevents *KRAS^G12V^*-induced hemocyte overproliferation. The ratio will be 0:1 if silencing of *GeneX* is completely lethal to the flies or has no effect on *KRAS^G12V^*-induced lethality. In step 2, we evaluated and confirmed the positive hits from step 1 by examining the hemocyte number at the third-instar larval stage (Fig. 4A).

After screening ∼3000 genes that are expressed in the hemocytes based on our unpublished hemocyte RNA sequencing (RNA-seq) data, we identified 24 genes that could completely rescue the lethality and hemocyte overproliferation of our *KRAS^G12V^* leukemia model at 25°C (Fig. 4B). All of these 24 genes are evolutionarily conserved from flies to humans [based on Drosophila RNAi Screening Center (DRSC) Integrative Ortholog Prediction Tool (DIOPT) score]. These genes encode proteins that are involved in transcription, cell cycle regulation, DNA replication and repair, signaling transduction, vesicle transport and vacuolar ATPase assembly (Fig. 4B). Numbers of hemocytes per third-instar larva were counted for gene silencing of each of these 24 hits, by itself and within the genetic background of the *KRAS^G12V^* leukemia fly model. Data were obtained for two independent RNA interference (RNAi) lines for each of the genes to ensure that none of the findings were due to off-target effects (Table S1). Consistent results were obtained for all 24 genes across both strains. We found that silencing any of these 24 genes could completely rescue the *KRAS^G12V^*-induced hemocyte overproliferation (Table S2).

### *Drosophila HIF1A* and *ARNT* orthologs *sima* and *tgo* are required for the survival of *KRAS^G12V^*-induced leukemia cells

**Fig. 5.**
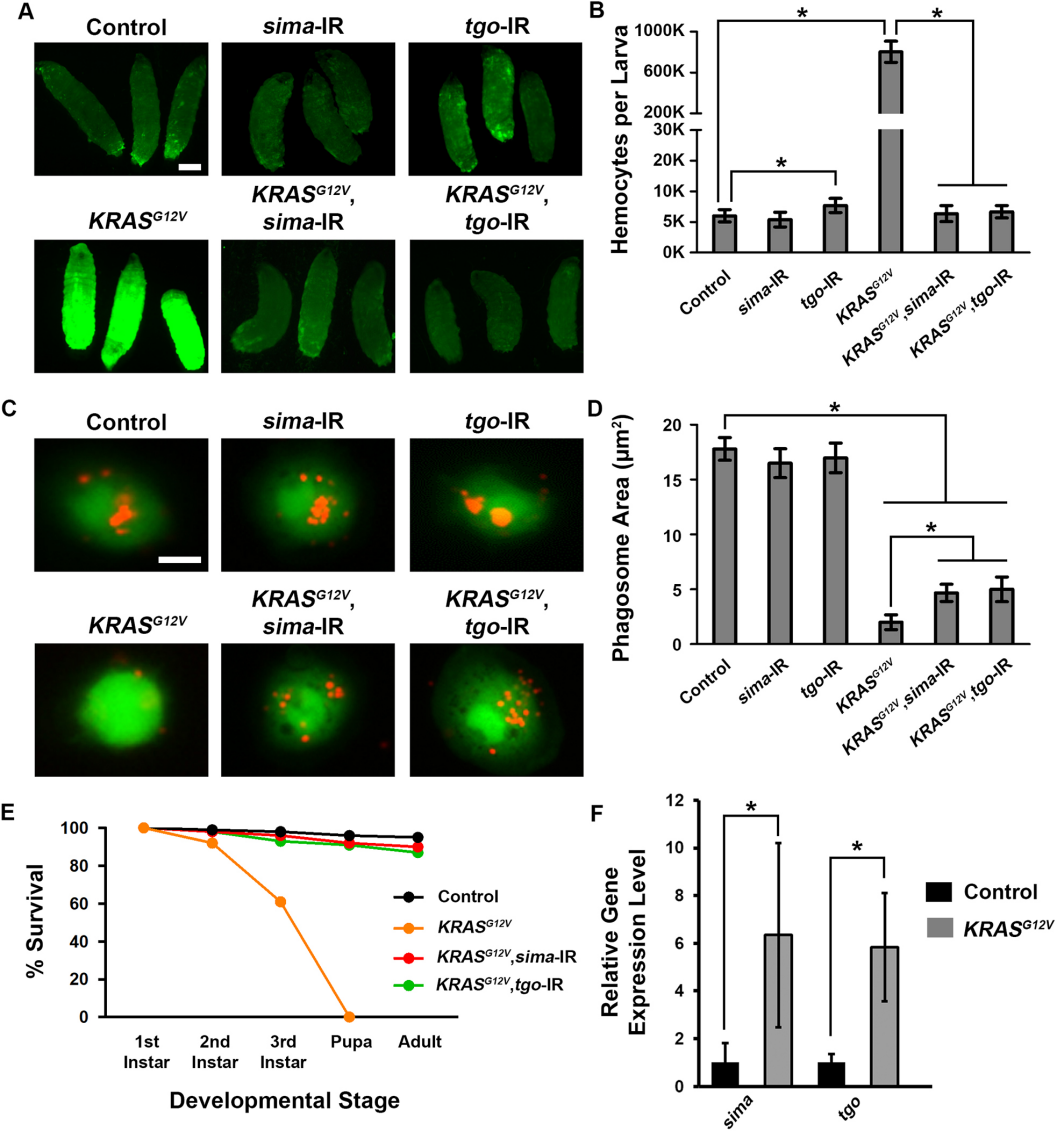
**Silencing *Drosophila* HIF1 complex gene homologs *sima* and *tgo* rescues *KRAS^G12V^*-induced hemocyte overproliferation, immune deficiency and pupal-stage developmental lethality.** (A) Left upper and lower panels show control (*Hml-Gal4, UAS-GFP*) and *KRAS^G12V^* (*Hml-Gal4, UAS-GFP; UAS-KRAS^G12V^*) third-instar larvae, respectively, in which all hemocytes express GFP (green fluorescence). Middle and right panels illustrate the effects of silencing *HIF1A* homolog *sima* (sima-IR) and *ARNT* homolog *tgo* (tgo-IR), respectively, in control (upper) and *KRAS^G12V^* (lower) third-instar larvae. Scale bar: 0.3 mm. (B) Quantification of total circulating hemocyte numbers with and without silencing of *sima* or *tgo* gene expression in control and *KRAS^G12V^* third-instar larvae (*n*=6; results are presented as mean±s.d.; *P<0.05; Kruskal–Wallis H-test). (C) Left upper and lower panels show hemocytes (green) extracted from control and *KRAS^G12V^* third-instar larvae, respectively, co-incubated with fluorescent Dextran-tagged *S. aureus* (red). Middle and right panels illustrate the effects of silencing *HIF1A* homolog *sima* (sima-IR) and *ARNT* homolog *tgo* (tgo-IR), respectively. Scale bar: 10 μm. (D) Quantification of phagosome area with and without silencing of *sima* or *tgo* gene expression in control and *KRAS^G12V^* hemocytes (*n*=100; results are presented as mean±s.d.; *P<0.05; Kruskal–Wallis H-test). (E) Survival during development of control, *KRAS^G12V^* and *KRAS^G12V^* flies in which *sima* (sima-IR) or *tgo* (tgo-IR) were silenced in hemocytes. (F) Quantitative RT-PCR showing the relative expression level of *sima* and *tgo* in hemocytes from control compared to *KRAS^G12V^* flies (*n*=3 replicates in each group; results are presented as mean±s.d.; *P<0.05; unpaired Student's *t*-test).

Among the 24 hits identified from the *KRAS^G12V^* genetic screen, *sima* (also called *HIF-1A*) and *tango* (*tgo*) are highly conserved fly orthologs of mammalian hypoxia-inducible factor 1 subunit alpha (*HIF1A*) and aryl hydrocarbon receptor nuclear translocator (*ARNT*, also called *HIF1B*), respectively. HIF1A and ARNT are transcription factors critically involved in the response to hypoxia (Dengler et al., 2014). Hemocyte-specific silencing of either *sima* or *tgo* by itself did not affect hemocyte proliferation, but silencing *sima* or *tgo* in the *KRAS^G12V^* leukemia model background completely restored the number of hemocytes down to wild-type levels (Fig. 5A,B). Next, we tested whether silencing *sima* or *tgo* could also rescue hemocyte phagocytosis function. Indeed, both were able to rescue the *KRAS^G12V^*-induced hemocyte phagocytosis defect, while silencing of *sima* or *tgo* alone did not affect phagocytosis (Fig. 5C,D). Further, we found that silencing of *sima* or *tgo* could completely rescue the lethality caused by *Hml*-*Gal4*>*KRAS^G12V^* at 25°C at each developmental stage compared to control flies (Fig. 5E). Concurrently, we observed significantly increased gene expression levels for *sima* and *tgo* in fly hemocytes carrying human *KRAS^G12V^* (Fig. 5F).

### A drug screen using the *KRAS^G12V^* leukemia fly model identifies echinomycin as an effective hit

**Fig. 6.**
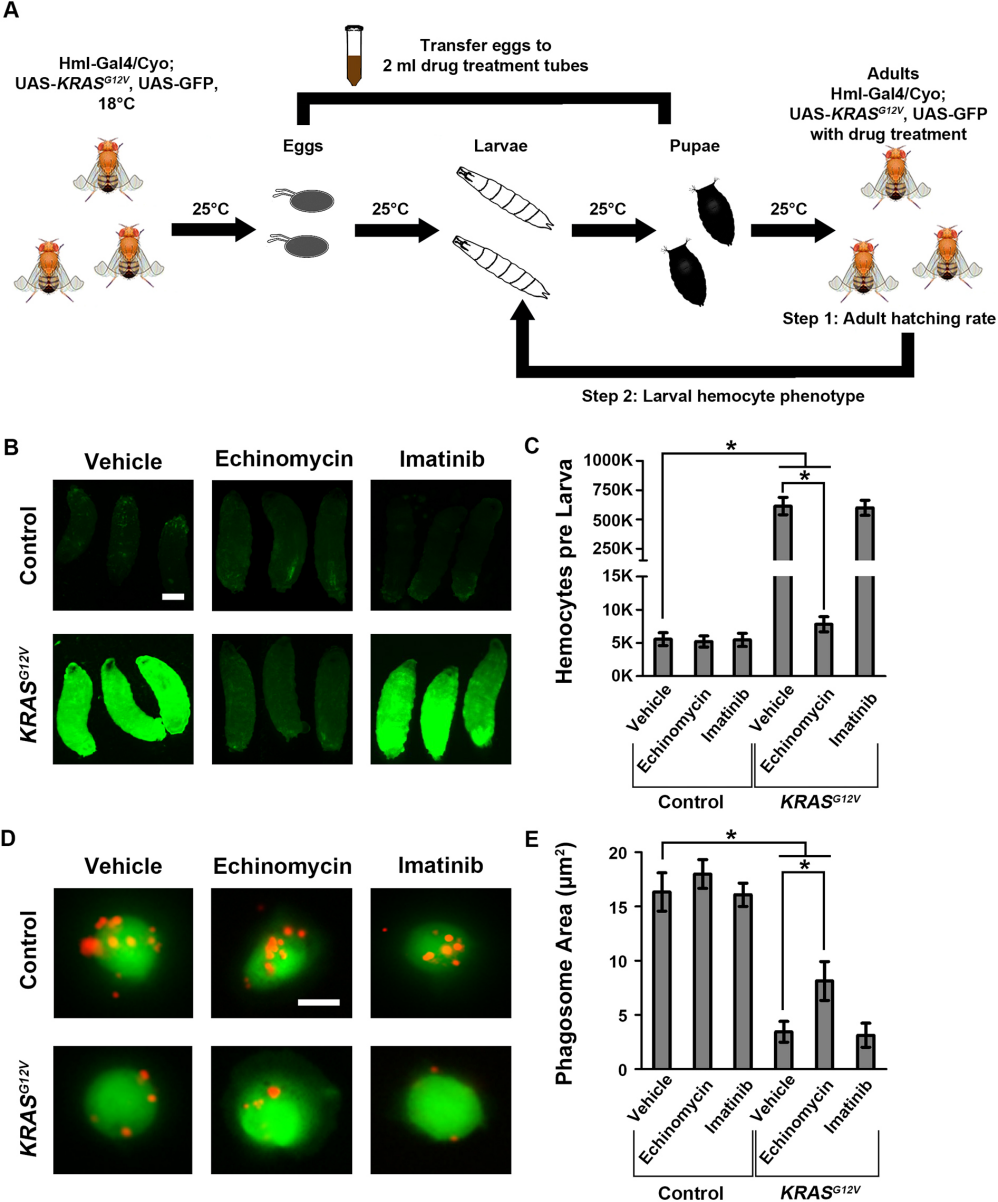
**Echinomycin reverses *KRAS^G12V^*-induced hemocyte overproliferation and immune deficiency.** (A) Schematic illustration of the approach used for the *KRAS^G12V^ Drosophila* leukemia model drug screen (see Materials and Methods section for a detailed description of rationale, strategy and procedure). (B) Left upper and lower panels show control (*Hml-Gal4, UAS-GFP*) and *KRAS^G12V^* (*Hml-Gal4, UAS-GFP; UAS-KRAS^G12V^*) third-instar larvae, respectively, in which all hemocytes express GFP (green fluorescence). Middle and right panels show the effects of treating larvae with echinomycin and imatinib, respectively. Scale bar: 0.3 mm. (C) Quantification of total circulating hemocyte numbers without (vehicle) or with echinomycin or imatinib treatment in control and *KRAS^G12V^* third-instar larvae (*n*=6; results are presented as mean±s.d.; *P<0.05; Kruskal–Wallis H-test). (D) Left upper and lower panels show hemocytes (green) extracted from control and *KRAS^G12V^* third-instar larvae, respectively, co-incubated with fluorescent Dextran-tagged *S. aureus* (red). Middle and right panels illustrate the effects of treatment with echinomycin and imatinib, respectively. Scale bar: 10 μm. (E) Quantification of phagosome area in control and *KRAS^G12V^* hemocytes with vehicle, echinomycin or imatinib (*n*=100; results are presented as mean±s.d.; *P<0.05; Kruskal–Wallis H-test).

In parallel with the large-scale genetic screen, we designed and performed a small-scale drug screen to identify compounds capable of rescuing the lethality of the *KRAS^G12V^* leukemia model at 25°C (Fig. 6A). We first collected eggs from the *Hml*-*Gal4*>*KRAS^G12V^* leukemia model and transferred ten fertilized eggs to each drug treatment vial, which contained a concentration of 0.5 μM drug in semi-liquid soft-gel food. When the fertilized eggs had hatched into larvae, they were fed on the drug-containing food. The drug treatment vials were kept at 25°C for 10 days to count the number of adult flies that eclosed in each vial. If a drug is completely lethal to the flies or has no effect on inhibiting *KRAS^G12V^*-induced hemocyte overproliferation, all flies would die before eclosion to adults. If a drug inhibits the *KRAS^G12V^*-induced hemocyte overproliferation, this will be evident in the number of eclosed adult flies.

We tested a collection of 34 chemical inhibitors (Table S3) available at our laboratory. This drug screen identified echinomycin to be the only drug among the 34 compounds that could completely rescue the lethality of the *KRAS^G12V^* leukemia fly model at 25°C (Fig. 6B,C). We also found that echinomycin could partially rescue the reduced phagocytic activity of *KRAS^G12V^* hemocytes (Fig. S3). Unexpectedly, the protein-tyrosine kinase inhibitor imatinib, inhibiting BCR-ABL, which can induce constitutive active downstream targets of oncogenic RAS (Cilloni and Saglio, 2012), failed to rescue both the *KRAS^G12V^* leukemia model adult-stage mortality (Fig. S3) and the reduced phagocytic activity of *KRAS^G12V^* hemocytes (Fig. 6B-E).

Echinomycin is a known inhibitor of the DNA-binding activity of HIF1A and HIF1B proteins (Kong et al., 2005). The identification of echinomycin as an effective drug for our *KRAS^G12V^* leukemia model further demonstrates the importance of the hypoxia signaling pathway in the *KRAS^G12V^*-induced leukemia phenotype. By identifying both the genetic factors and their inhibitors from parallel genetic and drug screens, our findings also show how effective the *Drosophila KRAS^G12V^* leukemia model can be in both types of screening platform in RAS-associated cancer studies.

### Echinomycin reduces the viability of human leukemia cells carrying oncogenic *RAS* mutations

**Fig. 7.**
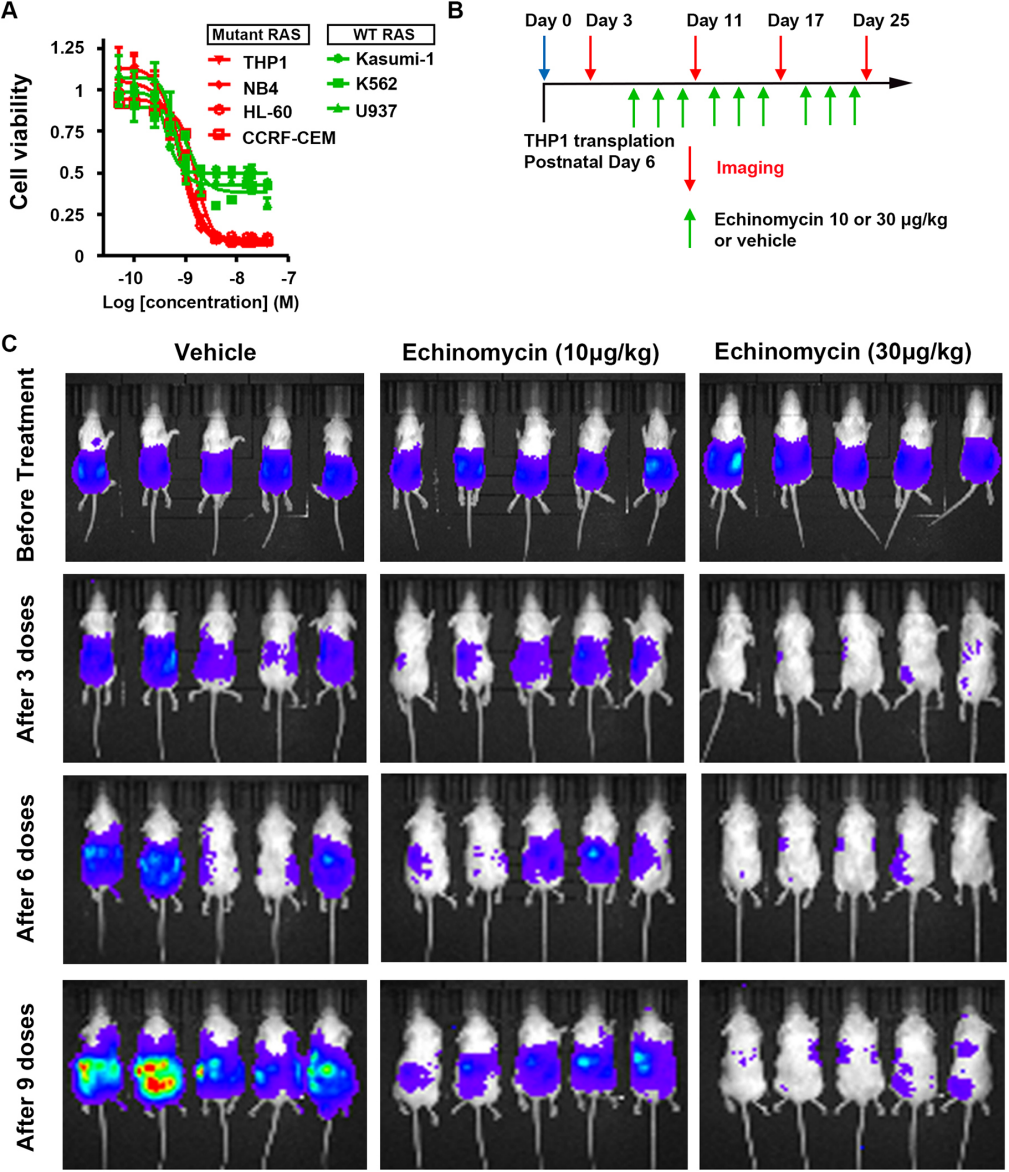
**Echinomycin reduces human leukemia cell viability and proliferation.** (A) Cell viability of human leukemia cell lines following echinomycin treatment. Human leukemia cell lines were treated with echinomycin for 48 h at the indicated concentrations. Cell viability was then determined by incubating cells overnight with MTT tetrazolium salt (Sigma-Aldrich). Experiments were performed in triplicate. Red, leukemia cell lines carrying *RAS* mutations; green, leukemia cell lines in which *RAS* is not mutated. (B) Schematic illustration of THP1 cell (human leukemia cell line carrying *NRAS^G12D^*) intrahepatic transplantation to generate mouse xenograft models, and time course of echinomycin treatments and diagnostic bioluminescence imaging to monitor THP1 cell transplant growth. (C) Bioluminescence imaging of mice engrafted with human THP1 leukemia cells bearing *NRAS^G12D^* mutation. Mice were treated every other day with 10 μg/kg or 30 μg/kg echinomycin (as indicated) or vehicle control and were imaged after every third dose.

To validate our findings from the *Drosophila KRAS^G12V^* leukemia model, we tested the effectiveness of echinomycin treatment in a panel of human leukemia cell lines, some of which carried oncogenic *RAS* mutations and other non-*RAS* mutations (Fig. 7A). We found that echinomycin treatment more potently reduced cell viability in the four oncogenic *RAS* cell lines [THP1 (*NRAS^G12D^*), NB4 (*KRAS^A18D^*), HL-60 (*NRAS^Q61L^*) and CCRF-CEM (*KRAS^G12D^*); Fig. 7A, red] than in the three leukemia cell lines without oncogenic *RAS* mutations (Kasumi-1, K562 and U937 leukemia cell line; Fig. 7A, green). Only ∼10% of *RAS*-driven leukemia cells remained viable after exposure to 4 nM echinomycin for 48 h, whereas ∼50% of non-*RAS* involved leukemia cells remained viable (Fig. 7A). This finding both validated the effect of echinomycin on attenuating the *Drosophila KRAS^G12V^* leukemia model in human cells and expanded the effective range of echinomycin to a broader range of oncogenic *RAS* mutations.

### Echinomycin inhibits human *NRAS^G12D^* leukemia cell proliferation in a mouse xenograft model

To further validate the effectiveness of echinomycin on leukemia cells induced by oncogenic *RAS* mutations in a mammalian *in vivo* model, we examined the effect of echinomycin administration on the growth of transplanted THP1 leukemia cells carrying oncogenic *NRAS^G12D^* (alone with a luciferase expression vector) in a xenograft mouse model (Fig. 7B). At day 3 post-transplantation and prior to drug administration, THP1 cell growth was readily apparent using live bioluminescence imaging of luciferase (Fig. 7C). Echinomycin treatment involving doses of either 10 μg/kg body weight or 30 μg/kg body weight effectively suppressed *in vivo* growth of human THP1 leukemia cells, with a clearly dose-dependent pattern, because higher dose treatment almost completely suppressed leukemia cell growth (Fig. 7C). These observations further validated that echinomycin can effectively suppress oncogenic *RAS*-induced leukemia cell growth in mammals, and further illustrated the value of the *Drosophila* leukemia model to identify this promising targeted drug to treat oncogenic *RAS*-induced cancer phenotypes.

## DISCUSSION

In this study, we generated a new *KRAS^G12V^* leukemia model in *Drosophila* by expressing human oncogenic *KRAS^G12V^* in fly hemocytes, which led to a nearly 100-fold increase in hemocyte proliferation and adult fly lethality. Using this easy-to-score phenotype, we designed highly efficient genetic and drug screens to identify genes and compounds able to inhibit oncogenic *KRAS*. Fortuitously, the parallel genetic and drug screens converged on the same pathways, as they identified both the key genes involved in the hypoxia pathway (*sima* and *tgo*), as well as the hypoxia inhibitor echinomycin as potent oncogenic *KRAS* antagonists. This discovery has provided strong evidence supporting an essential role for the hypoxia pathway in the oncogenic *KRAS*-induced cancer phenotype and has identified echinomycin as a promising hypoxia-targeting drug.

The genetic screen identified *sima* and *tgo* as mediators of the *KRAS^G12V^* pathomechanism. The two genes encode the fly homologs of human *HIF1A* and *ARNT*, respectively. HIF activation is a well-established feature of cancer cell survival in solid tumors, as cells positioned in the tumor interior are subject to severe oxygen deprivation, i.e. hypoxia. Activated HIFs lead to changes in the microenvironment that support tumor survival and growth, such as angiogenesis (Bousquet et al., 2016; Talks et al., 2000). In addition to the role of HIFs in solid tumors, HIF-mediated signaling has been found to play a crucial role in leukemia (Deynoux et al., 2016; Szymczak et al., 2018; Wang et al., 2011b). In addition, activated KRAS has been shown to induce *HIF1A* and *ARNT* target gene expression in human colon cancer cells (Chun et al., 2010). Echinomycin treatment has demonstrated therapeutic effect on acute myeloid leukemia cells with TP53 mutation in xenograft mouse models (Wang et al., 2020), but our study has shown, for the first time, that echinomycin is effective for oncogenic RAS in *Drosophila*, mouse and human cell systems. Our data also indicated significantly increased expression of *sima* and *tgo* in human *KRAS^G12V^* hemocytes (Fig. 5F), suggesting that uncontrolled proliferation of hemocytes within the larval hemocoel (body cavity), in our *in vivo* screen, could produce a hypoxic environment in which *HIF* gene function and HIF pathway activation are essential for cancer cell survival. In this scenario, *HIF* gene function would sustain progression and maintenance of leukemia in the fly, ultimately leading to death during the pupal stage. Consistent with this interpretation, *HIF1A* or *ARNT* gene silencing in hemocytes completely rescued adult fly development (Fig. 5E). To our knowledge, this is the first time *HIF1* gene(s) have been identified in a *KRAS* genetic screen and illustrates the value of an *in vivo Drosophila* model expressing a human oncogene for genetic screening purposes.

Furthermore, our fly leukemia model can be directly applied in an *in vivo* drug screen (Aritakula and Ramasamy, 2008; Dar et al., 2012; Gladstone and Su, 2011; Markstein et al., 2014; Willoughby et al., 2013). Testing the effect of multiple drugs at multiple concentrations in fly larvae revealed echinomycin as a strong inhibitor of *KRAS^G12V^*-induced hemocyte overproliferation. Interestingly, echinomycin is an inhibitor of the HIF1 pathway (Kong et al., 2005; Wang et al., 2011a, 2014). It prevents HIF1 binding to DNA target sites and thus disrupts HIF1 activation of target genes in response to hypoxic stress. Remarkably, echinomycin treatment was as effective as silencing either of the *sima* or *tgo* HIF pathway genes (Fig. 6). Moreover, echinomycin showed no toxicity to the fly at a concentration that completely reversed the leukemia phenotype (Fig. S3). Thus, these results have demonstrated another unique advantage of *in vivo* drug testing in *Drosophila* to identify potentially beneficial anticancer compounds of low organismal toxicity. Furthermore, we showed that echinomycin was particularly effective in multiple oncogenic *RAS* human leukemia lines compared to non-*RAS* cell lines and demonstrated its effect in a mammalian *in vivo* model for oncogenic *RAS* (xenograft mouse model using human THP1 cells with oncogenic *NRAS^G12D^*; Fig. 7). In addition, our recent data showed that echinomycin also effectively inhibited HIF1A oncoprotein and regressed lung tumor cell growth based on multiple RAS cell lines, including NCI-H727 (*KRAS^G12V^*), NCI-H1944 (*KRAS^G13D^*) and Calu-1 (*KRAS^G12C^*) (Huang et al., 2021). Taken together, these findings demonstrate the potential of echinomycin in treating *RAS*-induced cancer phenotypes. These findings are in line with previous studies that showed that echinomycin suppressed the growth of multiple AML cell lines and T-lymphoblastic leukemia cell lines (Yonekura et al., 2013) and effectively treated a mouse Mll-Flt3 AML model (Wang et al., 2011b, 2014). However, our study provides the first *in vivo* evidence that echinomycin treatment can attenuate oncogenic *KRAS*-induced leukemia in both *Drosophila* and mouse xenograft models.

Functionally, the *KRAS^G12V^*-expressing hemocytes showed excessive proliferation, which ultimately resulted in complete mortality at the pupal stage (i.e. no adult flies emerged). Furthermore, they displayed impaired innate immune capabilities as evidenced by significantly reduced phagocytic activity (which could be partially rescued by HIF1 silencing; Fig. 5C,D), increased sensitivity to bacterial infection and reduced immune-related gene expression (Fig. 3D). These observations stand in contrast with previous reports that found that *Drosophila* strains carrying activated fly *Ras85D^G12V^* in hemocytes displayed no changes in phagocytosis activity compared to wild-type flies (Arefin et al., 2017). This possibly reflects the differential effects of human *KRAS^G12V^* versus fly *Ras85D^G12V^* when expressed in hemocytes. Furthermore, a closer look at the expanded number of hemocytes caused by *KRAS^G12V^*-induced overproliferation revealed a substantially altered composition of circulating hemocytes. Composition was notable for an expanded population of hemocytes that expressed Wg, an early hemocyte lineage surface marker (Fig. 2A), and a reduced P1^+^ plasmatocyte population, which is typically the most abundant and shows phagocytic activity (Fig. 2C). Interestingly, increased HIF1A has been shown to mediate cancer stem cell maintenance in leukemia, a highly proliferative cell type (Wang et al., 2011b, 2014). *KRAS^G12V^* expression also drove expansion of the normally very small population of Lz^+^ crystal cells (Fig. 2B), which contribute to melanization reactions linked to innate immunity (Stofanko et al., 2010; Williams, 2007). It is possible that a subgroup of the Wg^+^ early hemocyte population also expresses the more mature crystal cell and/or plasmatocyte markers, as it has been previously reported that oncogenic RAS can lead to reprogramming and dedifferentiation (Ischenko et al., 2013). Owing to antibody limitations (all used here are mouse monoclonal), we cannot rule out this possibility at this time. In a recent study of single-cell profiling of *Drosophila* blood, we identified new hemocyte cell types (Fu et al., 2020). We are currently using this information to investigate how *KRAS^G12V^* expression changes the composition of the hemocyte population.

Future mechanistic studies to elucidate the pathways underlying the differential susceptibility of oncogenic RAS leukemic cells to HIF inhibition by echinomycin will be of great interest. These studies could identify additional drug targets to inhibit HIF pathway signaling. Testing the efficacy of these and additional drug candidates, such as those shown to inhibit oncogenic KRAS and HIF pathways in a colorectal cancer screen (Bousquet et al., 2016), in treating oncogenic RAS in model systems like our fly are urgently needed to identify valuable treatment options in the clinic. In conclusion, the data presented here demonstrate the significant role of hypoxia signaling in oncogenic RAS leukemias and identify echinomycin as a viable candidate for treatment. They also establish the potential of our *Drosophila* model for use in conducting genetic screens and drug screens for human cancers.

## MATERIALS AND METHODS

### Fly stocks

Flies were reared on standard food at room temperature or at 18°C or 25°C (as indicated). *Drosophila* lines were obtained from the Bloomington Drosophila Stock Center or Vienna Drosophila Resource Center unless otherwise indicated (Table S3). In order to generate transgenic fly lines carrying *UAS-HsKRAS^G12V^*, we cloned *HsKRAS^G12V^* into the *pUAST-attB* vector, and the transgenes were introduced into a fixed chromosomal docking site by germ line transformation, to ensure that the *RAS* alleles were transcribed at precisely equivalent levels in hemocytes.

### *RAS^G12V^* leukemia model

The *Drosophila* model was generated through genetic crosses that ultimately yielded flies of genotype *Hml-Gal4, UAS-GFP* (second chromosome)*; UAS-HsKRAS^G12V^* (third chromosome). In such flies, *Hml-Gal4* drove oncogenic *UAS-Ras* and *UAS-GFP* transgene expression specifically in hemocytes.

### *Drosophila* genetic screen

A genetic screen was used to identify genes required for hemocyte cell viability/proliferation in a background of human *KRAS^G12V^* expression. The approach involved simultaneous *Hml-Gal4*-driven hemocyte-specific expression of *UAS-KRAS^G12V^* with a *UAS-*RNAi gene-silencing construct targeting a fly gene that is expressed in the hemocyte based on our unpublished RNA-seq data. Lethality for a given *UAS-*RNAi-targeted gene was determined by crossing homozygous *UAS-*RNAi transgenic male flies to females carrying the *Hml-Gal4* driver balanced over *CyO* (‘Curly O’) and homozygous for *UAS-KRAS^G12V^* and *UAS-GFP* (females were raised at 18°C, at which temperature the yeast-derived Gal4 protein is unstable and therefore ineffective at driving *UAS-KRAS^G12V^* expression to levels inducing pupal-stage lethality; adult females were shifted to 25°C for genetic crosses and all subsequent screening steps were performed at 25°C). Progeny were first screened for emergence of straight-winged adult flies, indicating that silencing of the given gene by RNAi expression in hemocytes had prevented *KRAS^G12V^*-induced pupal-stage death (curly-winged adult flies inherited the *CyO* balancer chromosome rather than the *Hml-Gal4* driver and therefore expressed neither *KRAS^G12V^* nor RNAi in hemocytes). In cases of straight-winged adult fly emergence, larval-stage progeny were then examined to determine the number of circulating hemocytes (all of which expressed GFP).

### Hemocyte counting

Circulating hemocytes from single wandering third-instar larvae grown at 25°C were collected in 20 μl Schneider's *Drosophila* medium (Gibco). Larvae were carefully opened from the rear dorsal aspect and circulating hemocytes were gently transferred into the medium without disturbing the lymph gland. To count hemocytes, 10 μl of this mix was added to a hemocytometer, and GFP^+^ cells were counted using fluorescent microscopy. Hemocytes from six individual larvae of each genotype were counted using a hemocytometer.

### Quantitative RT-PCR

Circulating hemocytes were collected from wandering third-instar larvae. Total RNA was extracted using TRIzol LS Reagent (Thermo Fisher Scientific) according to the manufacturer's instruction. Briefly, 250 µl hemocytes were lysed with 750 µl TRIzol LS Reagent, then RNA was extracted with chloroform and precipitated with isopropanol. RNA was washed with 75% ethanol and finally solubilized in TE buffer. The concentration and quality (A260/A280) of RNA was checked by a Nanodrop 2000 spectrophotometer (Thermo Scientific). Complementary DNA was synthesized using a High-Capacity cDNA Reverse Transcription Kit (Thermo Fisher Scientific). Quantitative RT-PCR of immune response genes was performed with Power SYBR Green PCR Master Mix (Applied Biosystems), and data were analyzed using the 2^-ΔΔCT^ method. Gene *rp49* (also called *RpL32*) was used as an internal control for normalization of gene expression levels.

### Flow cytometry

Circulating hemocytes were collected from ten and two wandering third-instar *Hml-Gal4, UAS-GFP* and *Hml-Gal4, UAS-GFP; UAS-KRAS^G12V^* larvae grown at 25°C, respectively. The size and granularity of collected live cells were immediately analyzed by BD FACS Calibur flow cytometry, and results were analyzed using FlowJo software (BD Biosciences).

### Hemocyte labeling

Circulating hemocytes were collected from wandering third-instar larvae. Cells were attached to slides at 29°C for 60 min. Cells were then fixed for 10 min in 4% paraformaldehyde in PBS, blocked with 0.5% Triton X-100 plus 5% bovine serum albumin (in 1× PBS) for 1 h, incubated with primary antibodies anti-P1 (a kind gift from Dr I. Ando, Biological Research Center, Szeged, Hungary; 1:1000), anti-4D4 (Developmental Studies Hybridoma Bank; 1:1000) or anti-Lz (Developmental Studies Hybridoma Bank; 1:1000) at 4°C overnight. The cells were then washed three times with 1× PBS, incubated with Cy3-conjugated goat anti-mouse antibody (Jackson ImmunoResearch; 1:2000), washed three times with 1× PBS, mounted and imaged using a Zeiss Axio Imager with ApoTome2.

### Hemocyte phagocytosis assay

Circulating hemocytes were collected from ten wandering third-instar larvae and incubated for 20 min in Schneider's *Drosophila* medium with 1 mg/ml *S. aureus* or *E. coli* bacterial cells labeled with pHrodo Red (Thermo Fisher Scientific). Hemocytes were then fixed for 10 min in 4% paraformaldehyde in PBS. Confocal imaging was performed using a Zeiss ApoTome.2 microscope and a Plan-Apochromat 20×/0.8 NA air objective. For quantitative comparisons of fluorescence intensity between samples, we selected an imaging setting that avoided oversaturation and used it across all image collection for comparative analysis. ImageJ Software Version 1.49 was used for image processing.

### *Drosophila* drug treatment

A total of 34 compounds (both commercially available and proprietary; Table S3) were included in the pilot drug screen, including those targeting mTOR, AKT, AMPK and hypoxia signaling pathways. *Drosophila* food was ground to a powder-like consistency, and ∼2 mg was dispensed into a 2 ml tube. Drug was added to the food to a concentration of 0.5 μM. Ten eggs were transferred to each tube and maintained on drug-supplemented food at 25°C until growth and development to adult flies. Hemocytes were then collected for analysis in wandering third-instar larvae of those hatched flies.

### Transplantation of larval hemocytes into adult flies

Hemolymph samples containing circulating hemocytes were collected from third-instar larvae of control flies. Hemocytes from control (*Hml-Gal4, UAS-GFP*) and *KRAS^G12V^* (*Hml-Gal4, UAS-GFP; UAS-KRAS^G12V^*) larvae were collected in ice-cold Schneider's *Drosophila* medium. Hemocytes were centrifuged and resuspended in the same medium at 2.5×10^6^ cell/ml. A newly emerged wild-type male fly was anesthetized with CO_2_, then 200 nl of hemocyte cell suspension was injected into the abdomen using a Nanoject II injector (Drummond Scientific) fitted with a glass capillary prepared on a micropipette puller (Narishige PN-30). Hemocytes from each larval genotype were transplanted into 50-100 adult flies of wild-type genetic background. Transplant-bearing flies were then transferred to fresh food and maintained for 2 h (day 1) or 4 days prior to analysis. For analysis, hemolymph samples collected from three files were pooled in 20 µl Schneider's *Drosophila* medium, and GFP^+^ hemocytes were counted using a hemocytometer.

### Infection and survival assays

Experiments were performed as described previously (Shokal and Eleftherianos, 2017). *P. luminescens* was cultured in sterile Luria–Bertani (LB) broth for 18-22 h at 30°C on a shaker (220 rpm). The bacteria were then pelleted by centrifugation, washed and re-suspended in 1× sterile PBS. The concentration of bacteria for fly infections was adjusted to optical density (OD, 600 nm) 0.1 using a spectrophotometer. Seven- to 10-day-old adult male flies were anesthetized with CO_2_, then 18.4 nl bacterial suspension or sterile 1× PBS (injury control) was injected into the thorax using a Nanoject II apparatus (Drummond Scientific) equipped with a glass capillary prepared on a micropipette puller. Forty flies of each genotype were infected and subsequently maintained at 25°C. Survival was monitored for 48 h following injection.

### Human leukemia cell viability assay

The human leukemia cell line CCRF-CEM was purchased from American Type Culture Collection (ATCC), and all other cell lines were maintained in Y.L.’s laboratory. The status of *KRAS* of U937 (*KRAS* wild type) and NB4 (*KRAS^A18D^*) was obtained from Babij et al. (2011). Information on the other cell lines was obtained from ATCC, including Kasumi-1 and K562 (both *KRAS* wild type), THP1 (*NRAS^G12D^*), HL-60 (*NRAS^Q61L^*) and CCRF-CEM (*KRAS^G12D^*). In total, four human cell lines with *KRAS* mutations and three lines with wild-type *KRAS* were assayed. Cell viability was determined using Cell Proliferation Kit I (MTT; Sigma-Aldrich) according to the manufacturer's instructions. Briefly, cells were seeded at a density of 8×10^4^ cells/well in a 96-well plate and maintained at 37°C for 48 h in the presence of vehicle (control) or echinomycin at increasing concentrations. Next, 10 μl MTT was added to each well and the plate incubated for a further 4 h in a humidified incubator. Then, 100 μl of solubilization solution was added to each well and the plate was kept at 37°C overnight. Dye was quantitated using a scanning multi-well spectrophotometer (BioTek Instruments).

### Human leukemia xenograft

Initially, 21 *Nod.Scid.Il2rg^0^* [NOD scid gamma (NSG)] mice, postnatal day 6, received intrahepatic transplants of 10^6^ lentiviral luciferase transduced human THP1 *NRAS* mutant leukemia cells in ice-cold PBS as previously reported (Wu et al., 2016). Human leukemia cell engraftment was confirmed 3 days later by live bioluminescence imaging using a Xenogen IVIS Spectrum Imaging System (Caliper Life Sciences). Beginning on postnatal day 6, mice received intraperitoneal injections of vehicle, or 10 μg/kg or 30 μg/kg echinomycin (seven mice in each treatment group) every other day for a total of nine injections. Two mice in the 30 μg/kg echinomycin group did not survive the first three doses of treatment; therefore, results for only five mice in each group were included in further analysis. Bioluminescence imaging was performed after every third dose. For imaging, mice received IP d-luciferin (K^+^ salt, 150 mg/kg, Caliper Life Sciences) and 10 min later were anesthetized with isoflurane. Imaging results were analyzed using Living Image software (PerkinElmer). Signal values (photons/s/cm^2^/sr) were obtained for regions of interest, and quantitative comparisons across control and treatment groups were based on mean bioluminescence values.

### Statistical analysis

Statistical tests were performed using PAST.exe software. Data were first tested for normality by using the Shapiro–Wilk test (a=0.05). Normally distributed data were analyzed either by unpaired Student's *t*-test (two groups) and Bonferroni comparison to adjust the *P*-value or by a one-way ANOVA followed by a Tukey–Kramer post-test for comparing multiple groups. Data without a normal distribution were analyzed by either a Mann–Whitney test (two groups) and Bonferroni comparison to adjust *P*-value or a Kruskal–Wallis H-test followed by a Dunn's test for comparisons between multiple groups. Statistical significance was defined as *P*<0.05.

### Ethics approval

This study was approved by the University of Maryland School of Medicine Institution Review Board.

This article is part of a collection ‘The RAS Pathway: Diseases, Therapeutics and Beyond’, which was launched in a dedicated Special Issue guest edited by Donita Brady and Arvin Dar. See related articles in this collection at https://journals.biologists.com/dmm/collection/5089/The-RAS-Pathway.

## Supplementary Material

Supplementary information
