# Supplementary material for: Pharmacological or genetic inhibition of hypoxia signaling attenuates oncogenic RAS-induced cancer phenotypes
Source: Dis Model Mech. 2021 Nov 19;15(2):dmm048953. doi: 10.1242/dmm.048953 (PMC8617310; doi:10.1242/dmm.048953)
Supplement: Supplementary information [file dmm-15-048953-s1.pdf]

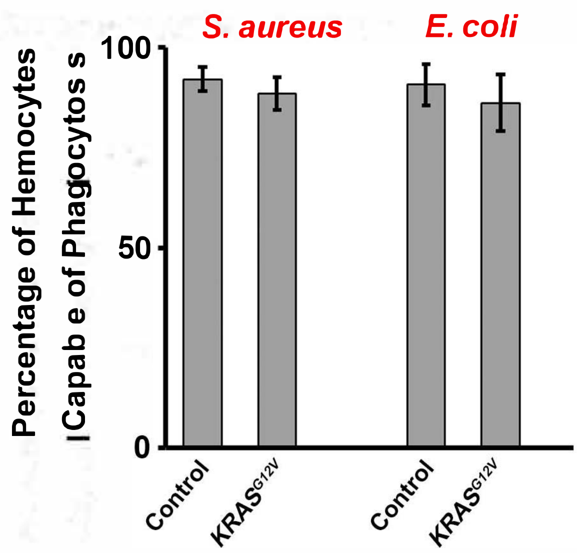

Fig. S1. Human *KRAS*^{G12V} transgene expression does not cause reduction in percentage of hemocytes capable of phagocytosis. Quantification of percentage of hemocytes capable of phagocytosis in control and *KRAS*^{G12V} hemocytes co-incubated with fluorescent pHrodo Red-tagged *S. aureus* or *E. coli* (*n*= 4 replicates in each group, results are presented as mean ± SD; *P < 0.05).

Supplemental Figure 2

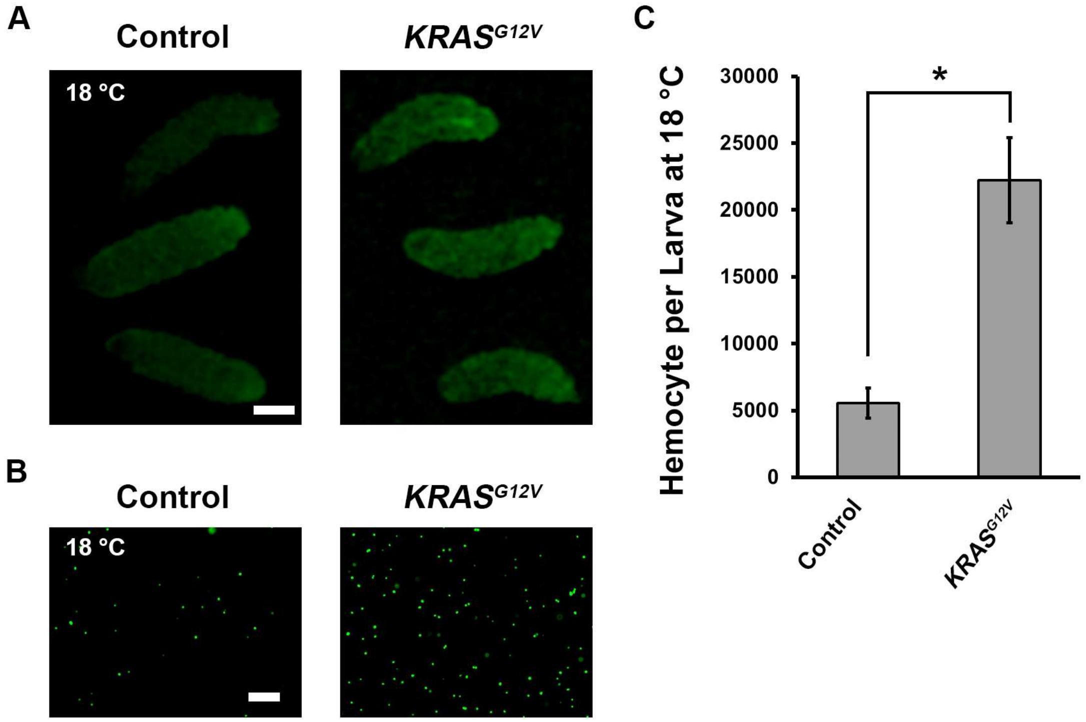

Fig. S2. Human *KRAS*^{G12V} mutant transgene drives hemocyte proliferation in *Drosophila* at 18 °C. (A) Transgenic (third-instar) larvae carrying hemocyte-specific *Hml-Gal4* driver directing expression of control and KRASG12V at 18 °C. Scare bar: 0.3 mm. **(B)** Hemocytes in hemolymph samples of equal volume extracted from control, and *KRAS*^{G12V} third instar larvae at 18 °C. Scale bar: 50 µm. **(C)** Quantification of total hemocytes per control and *KRAS*^{G12V} third-instar larvae at 18 °C (n=6, results are presented as mean ± SD; $^*P < 0.05$).

Disease Models & Mechanisms • Supplementary information

Supplemental Figure 3

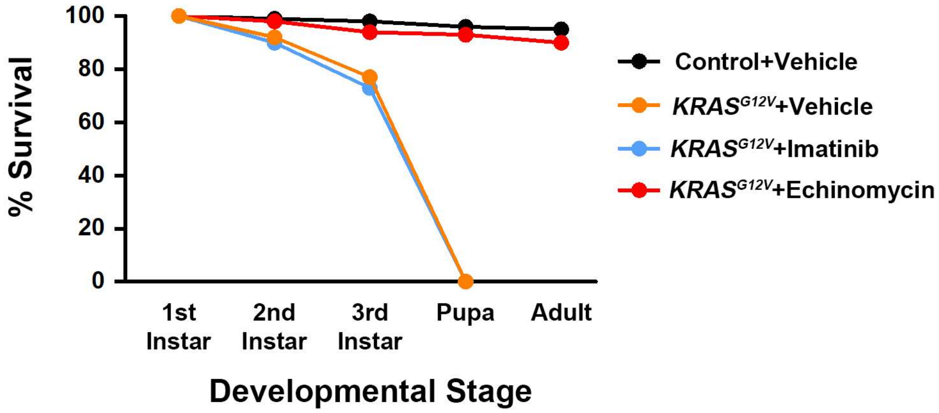

Fig. S3. Echinomycin reverses *KRAS*^{G12V}-induced pupal stage developmental lethality. Survival during development of control, *KRAS*^{G12V}, and *KRAS*^{G12V} flies in which Imatinib or Echinomycin doses used for the drugs added were treated in hemocytes.

Table S1. List of *Drosophila* transgenic lines for the positive hits used in the genetic screen.

#	*Drosophila* Gene	Source 1	Source ID	Source 2	Source ID
1	*Sima*	BDSC	33895	BDSC	26207
2	*tgo*	BDSC	26740	BDSC	38928
3	*nau*	BDSC	50607	BDSC	31899
4	*CG7504*	BDSC	34683	VDRC	108940
5	*wde*	BDSC	33339	VDRC	105719
6	*stg*	BDSC	36094	BDSC	29556
7	*CycE*	BDSC	38902	BDSC	29314
8	*babo*	BDSC	41585	BDSC	25933
9	*ncd*	BDSC	58144	VDRC	22570
10	*Blm*	BDSC	31331	BDSC	31330
11	*arr*	BDSC	31313	BDSC	31473
12	*Wnt5*	BDSC	28534	BDSC	34644
13	*Mkk4*	BDSC	35143	BDSC	42832
14	*CaMKI*	BDSC	26726	BDSC	35362
15	*nesd*	BDSC	58314	BDSC	35009
16	*frac*	BDSC	31577	BDSC	62941
17	*Syx1A*	BDSC	25811	VDRC	33112
18	*Syx16*	BDSC	25884	BDSC	51856
19	*Bet1*	BDSC	41927	BDSC	58269
20	*Rab1*	BDSC	27299	BDSC	34670
21	*Rab4*	BDSC	33757	VDRC	106651
22	*Rab8*	BDSC	27519	BDSC	34373
23	*Sec5*	BDSC	27526	BDSC	50556
24	*Vha100-1*	BDSC	26290	BDSC	57860

Table S2. Quantification of total circulating hemocyte numbers with and without silencing of 22 positive *Drosophila* gene expression in control and *KRAS*^{G12V} third-instar larvae.

#	*Drosophila* Gene	Hemocyte Number	
		by itself	with *KRAS* ^{G12V}
1	*sima*	5458	6367
2	*tgo*	7217	6429
3	*nau*	4652	4830
4	*CG7504*	4850	4512
5	*wde*	4092	5000
6	*stg*	4250	4876
7	*CycE*	3380	4966
8	*babo*	4300	4848
9	*ncd*	4520	5376
10	*Blm*	4720	5400
11	*arr*	4900	4580
12	*Wnt5*	5340	4600
13	*Mkk4*	4728	5316
14	*CaMKI*	5248	4800
15	*nesd*	4048	4944
16	*frac*	4250	5398
17	*Syx1A*	5440	4861
18	*Syx16*	5240	4932
19	*Bet1*	4900	4728
20	*Rab1*	4930	4516
21	*Rab4*	5300	4960
22	*Rab8*	4920	5440
23	*Sec5*	4970	5460
24	*Vha100-1*	4600	5066

Table S3. List of compounds used in the drug screen and their manufacturers.

#	Drug Name	Manufacturer
1	AGI-5198	Selleckchem
2	Pyrvinium	Sigma
3	Pantoprazole	Sigma
4	Metformin hydrochloride	Fluka
5	Rapamycin	LC laboratories
6	PKR inhibitor C16	Abcam
7	SAHA	Sigma
8	5-Aza-2'-deoxycytidine	Sigma
9	MB-3	Sigma
10	SGC0946	Sigma
11	GSK-LSD1	Cayman Chemical
12	Echinomycin	Sigma
13	GSK-1120212	Selleckchem
14	BKM 120	Selleckchem
15	Methylprednisolone	Sigma
16	Puromycin aminonucleoside	Sigma
17	Adramycin	Sigma
18	ARS-1620	MedChemExpress
19	BIX 01294	Sigma
20	Rho inhibitor I	Cytoskeleton
21	Paraquat dichloride hydrate	Sigma
22	Imatinib mesylate	Sigma
23	Pioglitazone	Cayman Chemical
24	Cyclosporin A	Sigma
25	Dexamethasone	Sigma
26	Akt1/2 kinase inhibitor	Sigma
27	2-Deoxy-D-glucose	Sigma
28	Selinexor	Sigma
29	Ivermectin	Sigma
30	Imatinib	Sigma
31	Dorsomorphin	Sigma
32	Ampkinone	Cayman Chemical
33	Simvastatin	Sigma
34	Carbamazepine	Sigma

Disease Models & Mechanisms • Supplementary information
